# Female Scrap Collectors in Qumbu, Eastern Cape, South Africa Through the Lens of Resilience Theory

**DOI:** 10.3390/healthcare14152398

**Published:** 2026-08-05

**Authors:** Mzukisi Xweso, Catherina Schenck

**Affiliations:** 1 Department of Social Work, Faculty of Community and Health Sciences, University of the Western Cape, Bellville, Cape Town 7535, South Africa; 2 Lifestyle Diseases Research Entity, Faculty of Health Sciences, North-West University, Mafikeng 2745, South Africa; 3 DSTI/NRF.CSIR Chair in Waste and Society, Centre for Interdisciplinary Studies of Children, Families and Society, University of the Western Cape, Bellville, Cape Town 7535, South Africa; cschenck@uwc.ac.za

**Keywords:** female scrap collectors, mental health, trauma, resilience theory, South Africa

## Abstract

**Highlights:**

**What are the main findings?**

- Perpetual insecurity and gendered vulnerability in informal workspaces.
- Enduring public degradation and its psychological toll on female scrap collectors.
- Experiences of persistent stress due to insufficient earnings.
- Informal relations with truck drivers that act as sites of economic exploitation and psychological insecurity.

**What are the implications of the main findings?**

- Establishment of NGO-led safe zones and provision of protective equipment is imperative to mitigate the occupational vulnerability of scrap collectors.
- Strengthening accessible, community-based mental health services through multidisciplinary collaboration is essential for psychosocial well-being and social justice.

**Abstract:**

Background/Objectives: Women working in the informal sector are frequently exposed to multiple layers of vulnerability and trauma, yet their mental health experiences remain insufficiently explored. Methods: This qualitative study examined the mental health implications of trauma experienced by female scrap collectors in Qumbu, Eastern Cape. Guided by resilience theory, the study explored how these women navigate and cope with the challenges associated with their daily work. A total of 13 female scrap collectors were selected through convenience sampling and participated in semi-structured interviews. The data were analysed thematically. Results: The findings revealed that women engaged in scrap collection face perpetual insecurity and gendered vulnerability in informal workspaces, enduring public degradation with significant psychological consequences, experience persistent stress due to insufficient earnings and navigate informal relations with truck drivers that act as sites of economic exploitation and psychological insecurity. These experiences may contribute to chronic stress, fear, feelings of worthlessness and mental exhaustion, which significantly affect their psychological wellbeing. Conclusions: Based on these findings, the study recommends the implementation of targeted psychosocial support interventions, such as trauma-informed counselling and peer support groups, to enhance coping mechanisms and strengthen resilience. Furthermore, a multidisciplinary facilitative healthcare approach and advocacy efforts aimed at addressing the mental health needs of female scrap collectors are essential for strengthening their agency, promoting psychosocial wellbeing and reducing their exposure to harm within the informal working environment.

## 1. Introduction

The informal economy plays a significant role in sustaining the livelihoods of millions of individuals globally, particularly within developing countries where formal employment opportunities remain limited. Within this sector, waste picking and scrap collection have emerged as critical survival strategies for individuals and households facing persistent socio-economic marginalisation [1,2,3]. In South Africa, waste pickers contribute substantially to environmental sustainability and recycling processes by recovering recyclable materials that would otherwise be disposed of in landfills [4]. Despite their vital contributions to the circular economy and broader waste management systems, waste pickers remain largely invisible within policy frameworks and labour protections, often operating under precarious and hazardous conditions [5]. Their work is characterised by instability, exposure to environmental hazards, and limited access to social protection, all of which collectively place them at heightened risk of both physical and psychological harm [6].

Women engaged in scrap collection experience compounded vulnerabilities arising from the intersection of gender inequalities and the precarious conditions associated with informal labour [7]. Gendered disparities within the informal economy disproportionately channel women into highly marginalised and exploitative forms of work, exposing them to labour segmentation, economic insecurity, occupational hazards and multiple forms of social vulnerability [8,9]. Female scrap collectors frequently navigate unsafe public spaces, endure long working hours, and face the social stigma associated with their occupation. The conditions inherent in this work are profoundly adverse [3], exposing workers to a range of traumatic experiences, including harassment, intimidation, verbal abuse and the persistent threat of violence from strangers and community members. While the existing scholarship has extensively examined the economic contributions and livelihood strategies of waste pickers, a significant gap remains in the literature concerning the psychological consequences of these occupational risks, particularly among women. Studies on informal work have largely prioritised structural poverty, labour precarity, and economic survival, with comparatively limited attention given to the mental health implications arising from sustained exposure to unsafe and hostile working environments.

According to Refs. [10,11], exposure to traumatic experiences is strongly associated with a range of adverse mental health outcomes. Within the informal waste-picking sector, the nature of labour exposes workers to conditions that may profoundly compromise their psychological wellbeing. Ref. [12] provide critical insights into the harsh realities faced by waste pickers, highlighting that, beyond physical hazards, workers may experience significant psychological symptoms that impose a substantial burden on their mental health. Furthermore, the continuous exposure to violence, fear, humiliation, and physical danger documented in other studies [5,13,14] contributes to chronic stress, emotional distress and persistent feelings of insecurity. Over time, such conditions may culminate in psychological outcomes such as anxiety, depression, emotional exhaustion and a diminished sense of personal worth. However, the lived experiences of female waste pickers who endure these traumatic conditions and the cumulative mental health burden associated with them remain largely underexplored within academic discourse. This invisibility is particularly concerning, given that informal workers often lack access to institutional support systems, including mental health services, occupational protections, and social assistance programmes that could mitigate the psychological impact of their work.

Against this backdrop, the present study seeks to contribute to the growing body of literature on informal labour by foregrounding the mental health implications of trauma among female scrap collectors. By centring the lived experiences of women operating within this marginalised sector, the study aims to illuminate the psychological burdens that are often obscured by predominantly economic narratives of informal work, which frequently neglect critical mental health dimensions.

The aim of this study is therefore to explore the mental health implications of trauma experienced by female scrap collectors in Qumbu, Eastern Cape, South Africa. Specifically, the study seeks to answer the following research question: *How do traumatic experiences encountered during scrap collection influence the psychological wellbeing of female scrap collectors in Qumbu?*

## 2. Theoretical Framework

**Figure 1:**
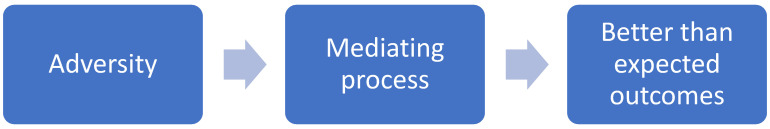
Resilience as a Process (Adapted from Ref. [17]).

This study is grounded in resilience theory, as advanced by Ref. [15], who conceptualises resilience as being built on a salutogenic approach, meaning the origins of how we view behaviour and social issues is on health and well-being. Ref. [16] therefore defines resilience as the “*capacity of individuals to navigate their way to the psychological, social, cultural and physical resources that build and sustain their well-being, and their individual and collective capacity to negotiate for these resources to be provided and experienced in culturally meaningful ways*”. In the South African context, characterised by persistent poverty and socioeconomic inequalities, resilience provides a valuable theoretical lens for understanding how individuals and communities respond to adversity. Ref. [17] defines resilience as “the multilevel processes that systems engage in to obtain better than expected outcomes in the face or wake of adversity”. This definition conceptualises resilience as a dynamic, multilevel and contextually embedded process rather than as a fixed individual attribute or a set of protective factors. Within this process, protective factors serve as mediating resources that facilitate positive adaptation and contribute to better-than-expected outcomes during or following adversity, rather than constituting resilience itself. According to Ref. [17], resilience theory comprises three interconnected components, namely the experience of adversity, the mediating resilience processes, and better-than-expected outcomes, as illustrated in Figure 1.

Collectively, these components provide a framework for understanding the contexts of vulnerability that affect individuals within the social dimensions of their lives, the mediating factors such as social support systems that enhance coping in the face of adversity, and the attainment of more adaptive or positive outcomes that reflect the capacity to recover and “bounce back”. Most importantly, for the purposes of this study, resilience theory offers a relevant framework for examining the mediating factors that shape the mental health outcomes of scrap collectors.

Ref. [18] provide a compelling alignment between resilience theory and the strengths-based perspective. Their argument enriches the position advanced by Ref. [15], who emphasises the importance of mediating processes also referred to as resilience processes or protective resources that enable individuals facing trauma and adversity to navigate challenges and achieve more adaptive outcomes. Within this framework, the strengths-based perspective [19] highlights the positive capacities that individuals bring to a given situation, including past experiences, talents and skills, which are essential resources that support a person’s ability to “bounce back” following adverse or traumatic experiences [18]. This theoretical alignment is particularly relevant to the present study, as it provides a lens through which to understand how female scrap collectors navigate adversity in the context of their informal work. Although their working conditions often expose them to traumatic experiences that adversely affect their mental health, these women simultaneously draw on personal and contextual strengths that enable them to cope, adapt and continue functioning within challenging environments. In this study, the primary focus is on the adversity encountered by female scrap collectors, as conceptualised within Ref. [15] resilience theory. Accordingly, the analysis foregrounds the structural and lived conditions that constitute adversity within their everyday working environments. Other dimensions of resilience theory, particularly mediating resilience processes and better-than-expected outcomes, were not used as predetermined coding categories during the initial thematic analysis. Rather, the findings were generated inductively from participants’ narratives, after which these components of resilience theory were applied deductively during the interpretation and discussion of the findings. This secondary theoretical interpretation enabled the researchers to contextualise the identified experiences of female scrap collectors within Ref. [17] resilience framework and to consider potential interventions and supportive measures that may strengthen resilience, enhance wellbeing, and improve their working conditions.

## 3. Materials and Methods

### 3.1. Study Approach and Design

This study benefited from the application of a qualitative approach [20], complemented by the strengths of an exploratory and descriptive research design. The nature of qualitative research provided an appropriate framework for examining the traumatic experiences of women engaged in scrap collection in Qumbu, as it enabled an expressive mode of inquiry through open-ended questions that allowed participants to articulate their experiences in depth [21]. Qualitative research facilitates the expansion and deepening of understanding by generating rich, contextualised data (Fouche et al., 2021) [20]. It is a form of inquiry that seeks to explore and provide deeper insights into real-world social problems (Korstjens & Moser, 2018) [22]. Rather than focusing on the collection of numerical data or the introduction of interventions, as is characteristic of quantitative research, qualitative research is particularly valuable for generating hypotheses and uncovering nuanced human experiences [21]. In this regard, the qualitative approach made it possible to solicit meaningful and contextually grounded information in response to the research question outlined in the introduction of this article. Such depth of understanding would have been difficult to achieve through a purely quantitative approach, which may have provided limited insight into how these women experience trauma and how such trauma is embedded within the context of their work.

### 3.2. Research Setting and Recruitment of Participants

**Figure 2:**
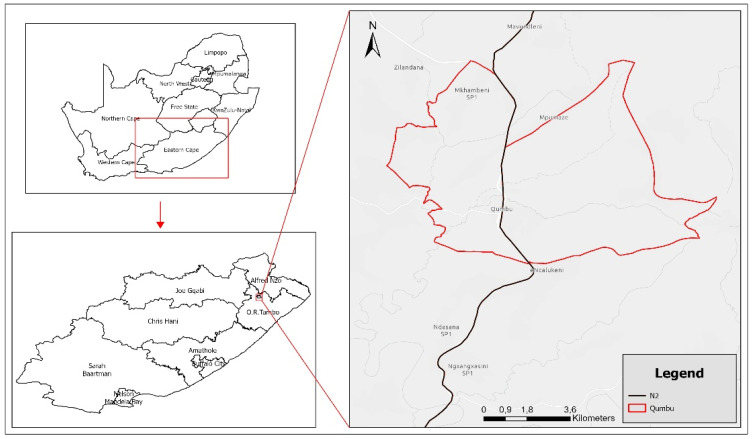
Map of the Qumbu study area, Eastern Cape. Source: North-West University, Geography Department.

The identification and recruitment of participants unfolded through a structured, two-stage process:

Initially, reconnaissance was conducted, involving observational surveys of sites where scrap collectors commonly congregate along the N2 highway. During this phase, the researcher traversed the highway, as depicted in Figure 2, and identified women waiting along the roadside within Qumbu town.

Subsequently, participant selection was carried out. Two distinct groups of women, situated approximately 5 km apart, were identified: Group 1 comprised six women, and Group 2 comprised seven. Convenience sampling, a non-probability technique [23], that involves selecting participants who are readily accessible and willing to participate, was employed. All women in the identified groups were available and consented to participate in the study. The inclusion criteria for the study were carefully established to ensure the appropriate and relevant selection of participants:

- Firstly, participants were required to be at least 18 years of age.
- Secondly, they had to have been engaged in scrap metal collection for a minimum period of one year. This requirement was considered essential, as individuals with limited experience in scrap metal collection may not yet have developed a comprehensive understanding of the challenges and hardships associated with this form of informal work.

Conversely, the exclusion criteria stipulated that women who had less than one year of experience in scrap metal collection were not eligible to participate in the study. This criterion ensured that the study included participants who possessed sufficient experiential knowledge of the realities and demands inherent in scrap collection activities.

**Figure 3:**
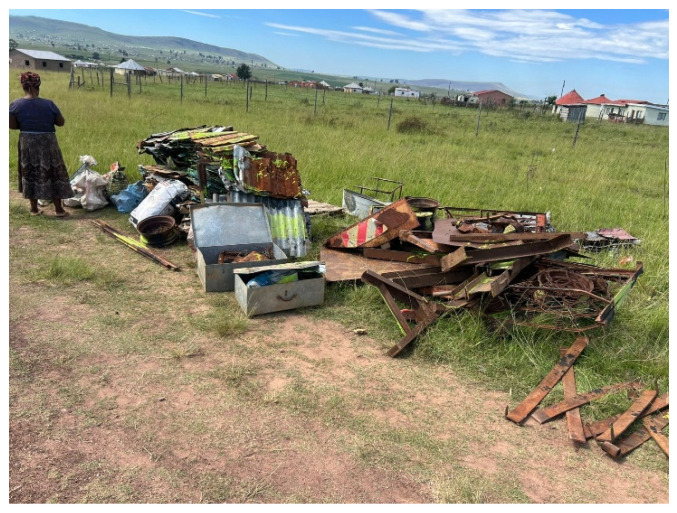
A photograph captured at the research site in Qumbu, along the N2 highway, illustrating the location where participants typically wait.

Within their physical working environments, the women who constitute the participants in this study present themselves in the manner illustrated in Figure 3 below.

Prior to engagement, comprehensive information about the study was shared to ensure informed participation.

### 3.3. Qualitative Data Collection

Data were collected through semi-structured individual interviews with participants who voluntarily consented to participate in the study. This method was selected because it enables participants to articulate their experiences in depth while allowing the researcher to obtain rich and detailed accounts relevant to the research question [20]. The use of individual interviews further ensured that participants could express themselves freely within a confidential and respectful research environment. The interviews were conducted at accessible sites along the N2 highway where the two groups of women engaged in scrap collection were commonly found. An interview guide was developed and utilised as an interview schedule to ensure consistency across interviews while still allowing flexibility for probing and follow-up questions where necessary. The interviews were conducted in isiXhosa, the primary language spoken by the participants, in order to facilitate clarity of communication, enhance participant comfort and ensure cultural and linguistic sensitivity during the data collection process. The interviews were conducted during a focused three-day data collection period.

A total of thirteen women engaged in scrap collection participated in the study. Each interview lasted no longer than 45 min. With the participants’ informed consent, the interviews were audio-recorded to ensure the accurate capture of participants’ narratives and to strengthen the credibility and trustworthiness of the data collected. The recordings were used solely for research purposes and handled with strict attention to confidentiality and ethical research practice. Data saturation was reached at the thirteenth interview, as subsequent interviews yielded recurrent narratives and no substantially new conceptual insights, thematic patterns, or variations relevant to the research objectives emerged. The researcher observed consistency across participants’ accounts regarding their experiences within their informal work, suggesting sufficient conceptual depth and thematic redundancy to adequately address the aims of the study.

### 3.4. Qualitative Data Analysis

The data generated through the semi-structured individual interviews were analysed using reflexive thematic analysis, guided by Braun and Clarke’s contemporary methodological framework, which recognises the researcher’s active and interpretive role in the construction of meaning within qualitative inquiry [24]. This analytical approach followed an iterative six-phase process that commenced with prolonged familiarisation with the data through repeated and careful reading of the interview transcripts. Such immersion enabled the researcher to develop a nuanced understanding of participants’ lived experiences, emotional expressions and contextual realities associated with scrap collection.

Thereafter, systematic coding was undertaken to identify significant segments of data relevant to the research objectives and questions. The coding process involved careful engagement with recurring meanings, patterns and conceptual relationships emerging across participants’ narratives. These codes were subsequently reviewed, compared and meaningfully clustered to generate preliminary themes reflecting broader interpretive patterns within the dataset. Attention was further given to points of convergence and divergence across participants’ experiences in order to strengthen analytical depth and conceptual coherence.

The preliminary themes were continuously reviewed, refined and reorganised to ensure that they accurately represented coherent, internally consistent and analytically distinct patterns of meaning. Given the interpretive nature of reflexive thematic analysis, reflexivity remained central throughout the analytic process. The researcher continuously engaged in critical self-reflection regarding how personal assumptions, disciplinary orientation within social science research, theoretical positioning and prior engagement with issues of marginalised population may have shaped data interpretation, coding decisions and theme development. This reflexive engagement contributed towards ensuring that the analytic interpretations remained grounded in participants’ narratives rather than predetermined assumptions.

The final stage of the analysis involved clearly defining and naming the themes, followed by the construction of an analytic narrative that systematically explains how the identified themes relate to the research questions, the theoretical framework and the broader objectives of the study. Through repeated engagement with the data and iterative refinement of interpretations, sufficient conceptual depth and analytical richness were achieved across the dataset. This rigorous and reflexive analytic process strengthened the interpretive credibility, coherence and depth of the qualitative findings. Table 1 presents the themes that emerged from this analytical process, which are subsequently discussed in relation to relevant literature and the theoretical framing of the study.

### 3.5. Ethical Considerations

All appropriate measures were undertaken to ensure full compliance with the ethical standards governing professional research in the social sciences. Ethical clearance for the study was granted by the Senate Research Ethics Committee of the University of the Western Cape, under ethics approval number HS20/5/27. Participants were informed that their involvement in the study was entirely voluntary and that their privacy and confidentiality would be rigorously safeguarded. Additionally, the scrap collectors were assured that the interviews would be conducted in a manner that would not interfere with their work, including times when they needed to collect or load scrap onto trucks, thereby minimizing any disruption to their livelihood activities.

### 3.6. Trustworthiness and Reflexivity

Several measures were undertaken to strengthen the trustworthiness, analytical rigor and interpretive credibility of the study. Credibility was enhanced through sustained engagement with participants during the data collection process, as well as prolonged immersion in the interview transcripts throughout the iterative phases of analysis. The researcher engaged in continuous comparative reflection across participants’ narratives to identify patterns of convergence, divergence and conceptual recurrence within the dataset. The application of reflexive thematic analysis further facilitated the critical interrogation, refinement and interpretive development of emerging themes, thereby enhancing analytic coherence and depth.

Dependability and confirmability were strengthened through the systematic maintenance of detailed analytic records, reflective engagement with the coding process and careful documentation of theme generation and refinement throughout the study. Reflexivity remained central to the analytic process, with ongoing critical consideration given to how the researcher’s disciplinary orientation, interpretive assumptions and engagement with issues of marginalisation and informal livelihoods may have shaped data interpretation. This process contributed towards ensuring that the findings remained closely grounded in participants’ lived experiences and narratives.

Furthermore, the incorporation of verbatim participant quotations enhanced interpretive transparency by illustrating the direct relationship between participants’ accounts and the analytic interpretations advanced in the study. Although formal triangulation procedures were not employed due to the exploratory and qualitative nature of the research design, interpretive rigor was reinforced through iterative engagement with the data, reflexive analytical practices and the consistent emergence of recurring conceptual patterns across participant interviews.

## 4. Results

### 4.1. Participants Characteristics

Table 1 presents the demographic and socio-economic characteristics of the study participants, offering insight into the profile of female scrap collectors in Qumbu who took part in the study.

The table indicates that the majority of participants are older women, predominantly between 48 and 60 years of age, with low levels of formal education, mainly limited to primary schooling. Many are single, widowed, or have never married and they support multiple dependents, underscoring substantial household responsibilities. Despite these challenges, the duration of engagement in scrap collection ranging from approximately 3 to 11 years reflects notable longevity, resilience and strong commitment. This sustained involvement further illustrates the trust these women place in scrap collection as a livelihood strategy for supporting their families, despite its limited income-generating capacity and the associated health risks and generally unsafe informal working conditions.

### 4.2. Key Themes Identified Through the Analysis of the Collected Data

#### 4.2.1. Theme 1: Perpetual Insecurity and Gendered Vulnerability in Informal Workspaces

In this study, participants consistently described a pervasive climate of fear linked to gendered vulnerability, personal safety and insecurity, which is inherent to the work of female scrap collectors. They reported experiencing heightened anxiety due to the absence of safe and designated waiting areas for trucks transporting them to Durban, leaving them exposed to potential harm while remaining at informal sites. This constant exposure to risk contributes to significant psychological strain, adversely affecting their wellbeing and overall mental health. Participants’ narratives indicate that prolonged periods spent in these spaces, often extending throughout the day and night, exacerbate feelings of vulnerability and heighten the risk of encountering criminal activity. The lack of secure shelter situates these women in a persistent state of precariousness, where the boundaries between work, rest and survival collapse, producing chronic stress and a sense of hyper-vigilance. The participants articulated the following experiences:

“We are constantly exposed to criminals because we spend both the day and night here, even sleeping in this space, which leaves us vulnerable”.

(P3)

“Some of the items we collect contain copper, which is very valuable when sold to scrap yards. However, some men come here and demand that we sell it to them at a much cheaper price. At times, they even take it from us by force and leave. Because of this, we must always remain vigilant for our safety”.

(P7)

“Sometimes when we travel to Durban to sell our scrap, we arrive on a Sunday and find that the places where we sell it, the buy-back centres, are closed. We then have to wait outside for the whole day and night until Monday. That situation is not safe for us, we can easily be robbed and when we see men walking around, we become afraid”.

(P12)

“You know, we are always worried because anything can happen to us here. We are exposed to many risks, and there is no one to protect us. We are here on our own”.

(P2)

In the above narratives, a 48-year-old female scrap collector (P12) reported that even upon reaching Durban, a large urban centre with potential for safer environments, such safety is largely unattainable. She described how they often remain outdoors at night, waiting for scrap dealers to open, observing men moving through these spaces, which heightens their sense of vulnerability. Although they do not report direct attacks, the persistent fear for personal safety generates uncertainty and anxiety, which can negatively affect their psychological state as they continue to engage in work that exposes them to potential violence at any moment. These accounts illuminate how informal workspaces operate simultaneously as sites of livelihood and zones of risk, where survival strategies are intertwined with continuous vigilance. This sustained anticipation of harm carries cumulative psychological implications, as prolonged exposure to unsafe environments can intensify stress responses, undermine feelings of personal security and influence daily coping behaviours.

#### 4.2.2. Theme 2: Enduring Public Degradation and Its Psychological Toll on Women Scrap Collectors

In the absence of safety in the spaces where they work, as highlighted in Theme 1, women waste pickers are subjected to persistent verbal hostility from members of the public. These interactions are not merely episodic but constitute a sustained pattern of social degradation that permeates their everyday working lives. The public nature of these encounters amplifies their impact, as the women are routinely exposed to judgement, ridicule and demeaning characterisations that strip them of dignity. Such treatment reflects deeply embedded societal stigma, wherein their livelihood is misrecognised and devalued, positioning them as objects of scorn rather than individuals engaged in legitimate economic activity.

“We see people all day who are insulting us here along the road. We do not stay peacefully here”.

(P8)

“People here think we are mad; they always call us with bad names. They do not know we want money and not that we are crazy”.

(P1)

“You know what people do? They do bad things to us like calling us things we do not like. It would have been better if we had a shelter where we can wait for the trucks and hide ourselves from the people we know, but we do not have a choice we must be here”.

(P13)

“I do not think people understand why we are doing the work we do…all they do is to give us names and looking down on us”.

(P10)

The accounts presented here reveal a profound erosion of psychological well-being driven by sustained exposure to verbal abuse and social invalidation. The mislabeling of these women as “mad”, as eloquently articulated by a 59-year-old female collector (P8) in the first account of theme 2, signifies a deeper process of dehumanisation, one that negates their agency and reinforces their marginal status within the social order. From a mental health perspective, such chronic experiences of disrespect and humiliation are likely to engender internalised stigma, emotional exhaustion and a diminished sense of self-worth. The relentless nature of these encounters fosters an environment of anticipatory distress, where the expectation of insult becomes embedded in their daily reality. Over time, this may manifest in heightened anxiety, social withdrawal and depressive symptoms, ultimately compromising their capacity to cope and function effectively within both their work and broader social contexts.

#### 4.2.3. Theme 3: Persistent Stress over Insufficient Earnings in Scrap Collection

In this study, women revealed that their physically demanding work exposes them not only to bodily strain but also to persistent stress over the insufficient earnings it generates. Coupled with the responsibilities of supporting dependents, this economic pressure places them under continuous strain, compelling them to collect scrap relentlessly, an effort that offers little relief for their physical well-being. The mental burden of constant worry is evident, as the intensity of the labour does not correspond to adequate financial reward, resulting in sustained anxiety over meeting family obligations, such as providing for household needs and ensuring children attend school. The participants articulated the depth of this stress, as reflected in the following sentiments:

“The money we earn from selling scrap is very little; however, one is forced to persist because there are no alternative means of surviving. There is no money in what we do at all”.

(P7)

“This work causes me significant stress. It is extremely physically demanding, yet the income is minimal and insufficient to meet basic needs”.

(P4)

“When I return from Durban, I would often experience severe stress, to the point of having headaches, because sometimes you come back with about R2000 ($117.23), for example. However, you still must pay R600 ($37.50) for the truck driver, leaving you with only R1400 ($82.06). What can you do with such a small amount of money? It does not even cover household needs. It is very stressing for us”.

(P10)

The findings suggest that the intensity of scrap collection undertaken by these women does not translate into meaningful financial earnings, rather, it is characterised by significant physical strain and heightened levels of stress, as they continually worry about income. This stress is closely linked to the fear that their families’ livelihoods are at risk, particularly in relation to meeting basic household needs. Participant 10’s account, which details the financial calculations of their earnings, further illustrates how limited income, after expenses, exacerbates this stress, as they return home with insufficient money while still facing pressing domestic demands. This reflects a persistent and burdensome reality in which stress becomes an enduring aspect of their daily lives.

#### 4.2.4. Theme 4: Informal Relations Between Scrap Collectors and Truck Drivers as a Site of Economic Violence and Psychological Insecurity

The participants further reveal that their interactions with truck drivers, who transport their scrap to Durban for sale to buy-back centres, are embedded within highly informal and unregulated arrangements. According to these women, as reflected in the account of a 38-year-old female in the final narrative of Theme 3, these relationships are characterised by the absence of contractual agreements, limited accountability, and a lack of identifiable information regarding those with whom they transact. Within this context, the same truck drivers—predominantly men—are entrusted with the transportation and prospective sale of the women’s collected materials. In some instances, these drivers forcibly appropriate the scrap and sell it independently, leaving the women stranded in Durban without access to the intended market. Such conditions create a precarious relational space in which trust is neither assured nor protected.

“These truck drivers give us wrong cell phone numbers and we struggle to get hold of them when we arrive in Durban, only to find out they have sold all the scrap we have collected”.

(P13)

“They told us we need to be in a separate transport because they have had talks with the traffic officers who have said that if they take people on the trucks, they will be taken to jail”.

(P5)

“We do not even know the names of these drivers, but the troubling thing is that it becomes difficult when they are selling our scrap. It is hard to trust them anymore, but we still trust that things will be fine one day”.

(P6)

The practices of the truck drivers as described by participants, particularly the forcible taking or unauthorised sale of their scrap materials, constitute a form of economic violence, as they deprive women of their primary means of livelihood. These encounters are further characterised by coercion and unpredictability, embedding a persistent sense of threat within everyday transactions. The gendered power imbalance inherent in these interactions heightens women’s vulnerability and limits their capacity to resist. From a mental health perspective, such conditions are likely to generate ongoing emotional strain, marked by fear, anxiety and insecurity. The anticipation of potential exploitation intensifies psychological distress and undermines their sense of stability. Over time, these experiences may erode trust and contribute to enduring trauma-related outcomes.

## 5. Discussion

Taken together, the findings enable a more explanatory application of Ref. [15] three-component resilience framework—adversity, mediating processes, and better-than-expected outcomes—by extending the interpretation beyond descriptive accounts and providing a theoretically informed understanding of how psychological wellbeing is negotiated under conditions of structural precarity. Rather than treating resilience as a trait or outcome that some individuals possess and others lack, the framework reveals resilience as a dynamic, multilevel process shaped by the interaction between structural conditions, relational resources and individual adaptive responses. Each theme identified in this study can be analytically located within this tripartite structure, thereby enabling resilience theory to function as an explanatory framework rather than a descriptive label.

With respect to the first component, adversity, the findings suggest that the experiences of female scrap collectors reflect what may be interpreted as cumulative adversity. This interpretation highlights how multiple, overlapping stressors interact and accumulate over time and across different life domains, thereby intensifying participants’ vulnerability and shaping their resilience processes. Crucially, the data demonstrate that each theme of adversity identified in this study does not operate in isolation. Gendered insecurity in informal workspaces (Theme 1) structurally undermines the psychosocial benefits that might otherwise arise from social recognition. Drawing on the complementary interpretative concept of ontological security [25], the findings further suggest that, when combined with chronic public degradation (Theme 2), these experiences may contribute to an erosion of individuals’ fundamental sense of safety, continuity, and stability of self. The co-occurrence of economic precarity (Theme 3) and relational exploitation by truck drivers (Theme 4) further closes off the pathways through which women might otherwise access protective resources, such as social trust, financial stability, or institutional recourse. This compounding dynamic explains why the psychological burden described by participants extends beyond what any single stressor would predict: it is the structural interlocking of adversity domains, rather than their individual severity, that generates chronic stress, hypervigilance, and emotional exhaustion. Resilience theory, applied in this explanatory mode, thus reveals the mechanisms through which structural inequalities translate into psychological harm.

The second component of Ref. [15] framework, the mediating processes, provides the most significant analytical leverage for understanding how women navigate adversity without necessarily overcoming it. The present study’s findings, while not centred on protective factors, surface implicit mediating processes within participants’ narratives that warrant theoretical attention. First, continued livelihood engagement itself functions as a mediating process: participants’ sustained presence in scrap collection, reflects an active, purposeful coping orientation grounded in caregiving responsibility. This aligns with Ref. [16] conception of resilience as the capacity to “navigate to” resources that are culturally meaningful, for these women, the resource being navigated toward is not wellbeing in an abstract sense, but the concrete capacity to sustain their dependants. Second, the data imply the presence of informal social cohesion within the two groups of scrap collectors identified in the study. Although participants did not explicitly describe these groupings as a source of psychosocial support, this pattern may represent an informal social process that warrants further investigation regarding its potential role in mitigating feelings of isolation and fear among female scrap collectors.

Regarding the third component, better-than-expected outcomes, Ref. [15] cautions against this individualistic reading, insisting that better-than-expected outcomes must always be assessed relative to the severity of the adversity encountered and the structural resources available. This study identifies the structural determinants of psychological possibility, rather than merely documenting suffering and offers an exploratory, contextually situated conceptual contribution that may contribute to the ongoing refinement of the resilience literature. Previous studies on waste pickers have largely framed psychological harm as a consequence of individual exposure to occupational hazards [12,26], while the present study’s findings focus on the interaction between adversity and the systematic denial of access to buffering resources, whether social recognition, economic security, or institutional protection. This theorisation moves the analytical frame from a deficit model focused on harm, toward a structural critique of the conditions that prevent women from exercising resilience more fully. In policy terms, this explanatory logic points not toward individual therapeutic interventions as primary responses, but toward the structural redesign of the informal waste economy to ensure that the mediating processes necessary for resilience—safe workspaces, regulated labour relations, accessible mental health support—are equitably distributed to those operating within it.

## 6. Study Strengths and Limitations

This study contributes to the growing body of scholarship examining the psychosocial dimensions of informal labour by foregrounding the lived experiences and mental health vulnerabilities of female scrap collectors within a rural South African context. In particular, the study provides contextually grounded insights into how structural precarity, gendered insecurity, public degradation and economic exploitation intersect to shape the psychological wellbeing of women engaged in informal waste work. In doing so, the study contributes to broader academic discussions concerning mental health, gendered informal labour and structural marginalisation within the Global South.

A key strength of the study lies in its qualitative and exploratory design, which enabled an in-depth engagement with participants’ lived realities and facilitated a nuanced understanding of the cumulative psychological strain associated with scrap collection. The application of resilience theory further provided a useful analytical lens for interpreting how women navigate adversity and sustain their livelihoods despite persistent insecurity and limited institutional support. By centring the voices of women operating within a marginalised and under-researched informal work environment, the study offers important contextual insights that are often overlooked within predominantly economically oriented discussions of waste work.

However, several limitations should be acknowledged. The study was geographically confined to Qumbu in the Eastern Cape Province of South Africa and focused exclusively on female scrap collectors, which limits the transferability of the findings to other informal waste work contexts and demographic groups. Furthermore, as an exploratory qualitative study based on a relatively small sample, the findings are intended to provide context-specific interpretive insights rather than broad generalisable conclusions. The study also did not seek to evaluate the effectiveness of mental health or policy interventions, and therefore the implications advanced should be interpreted within the scope and limitations of the research design.

Notwithstanding these limitations, the study provides an important foundation for future research examining the relationship between informal waste work, gendered precarity and psychological wellbeing. Future studies incorporating broader demographic groups, comparative contexts and interdisciplinary approaches may contribute towards a more comprehensive understanding of the mental health implications associated with informal labour across different settings.

## 7. Recommendations

Based on the findings of this exploratory qualitative study, several contextually grounded implications may be considered for supporting female scrap collectors operating within precarious informal work environments. The narratives presented by participants suggest the potential value of developing safer and more supportive working environments for women engaged in scrap collection. In this regard, community-based organisations and non-governmental organisations (NGOs) operating within the O.R. Tambo Municipality and surrounding areas could potentially explore the feasibility of establishing designated waiting spaces where scrap collectors may congregate while awaiting transportation to Durban for the sale of recyclable materials. Such spaces may contribute towards reducing exposure to insecurity, public hostility and environmental vulnerability. In addition, the provision of basic protective resources and safety support mechanisms may assist in mitigating some of the occupational risks associated with informal scrap collection activities.

The findings further highlight the importance of strengthening accessible and community-oriented psychosocial support initiatives for women operating within the informal economy. Although the present study was not designed to evaluate intervention effectiveness, participants’ narratives suggest that locally grounded mental health support services, including peer-support initiatives, counselling spaces and community-based coping interventions, may offer meaningful psychosocial support to women experiencing chronic stress and emotional strain linked to informal work conditions. Existing community mental health initiatives within the Eastern Cape, such as those operating in Mthatha and surrounding areas, may provide useful foundations for future outreach-oriented support strategies targeting geographically marginalised populations.

Furthermore, the findings point towards the potential relevance of collaborative and multidisciplinary approaches in responding to the psychosocial vulnerabilities experienced by female scrap collectors. Greater engagement between social workers, community development practitioners, public health professionals and local advocacy structures may contribute towards more holistic and context-sensitive responses to the challenges faced by women within informal waste economies. While broader structural interventions fall beyond the scope of this exploratory study, the findings nonetheless underscore the importance of recognising the interconnected relationship between informal labour conditions, gendered precarity and psychological wellbeing within marginalised communities.

## 8. Conclusions

In conclusion, the findings of this study indicate that women engaged in scrap collection in Qumbu experience significant psychological and emotional strain associated with structural exclusion, public degradation, economic precarity and gendered insecurity within informal work environments. The study further illustrates how trauma and mental health vulnerability are embedded within everyday survival practices, shaping women’s experiences of informal labour in complex and cumulative ways. While the findings are contextually situated and not intended for broad generalisation, they nonetheless contribute important insights into the intersection between gendered informal work, precarity and psychological wellbeing within marginalised communities.

From the perspective of resilience theory, the study demonstrates that women continue to navigate and endure adverse working conditions through ongoing adaptive survival practices despite persistent insecurity and limited structural support. The findings therefore suggest the importance of greater scholarly and practice-based attention to the psychosocial dimensions of informal waste work, particularly within under-researched rural contexts. Furthermore, the study highlights the potential value of context-sensitive and community-oriented support initiatives that may contribute towards strengthening psychosocial wellbeing and social inclusion among women operating within the informal economy. In this regard, the study contributes to broader discussions concerning social justice, gendered vulnerability and mental health within informal labour systems as similarly observed in other parts of the world.

## Figures and Tables

**Table 1:** Characteristics of female scrap collectors who participated in the study.

Participant ID	Age	Marital Status	Education	Number of Years Working as a Scrap Collector	Number of Dependants
P1	58	Separated	Grade 7	11 years	4
P2	60	Married	Grade 5	11 years	7
P3	59	Widowed	Grade 6	11 years	5
P4	50	Never married	Grade 6	11 years	4
P5	51	Married	Grade 6	10 years	5
P6	54	Never married	Grade 5	11 years	6
P7	55	Married	Grade 4	5 years	1
P8	59	Married	Grade 7	4 years	3
P9	48	Never married	Grade 11	3 years	3
P10	49	Staying with a partner	Grade 11	6 years	2
P11	38	Never married	Grade 6	3 years	2
P12	48	Never married	Grade 5	4 years	1
P13	55	Never married	Grade 5	4 years	2

## Data Availability

The data supporting the findings of this study are not publicly available due to the sensitive nature of human participant data. However, they may be obtained from the corresponding author, upon reasonable and appropriate request.
